# A Lecture on Poisoned Wounds, Delivered at the Philadelphia Medical Institute

**Published:** 1838-08-01

**Authors:** Thomas Harris

**Affiliations:** Surgeon U. S. N., and to the Pennsylvania Hospital


### A LECTURE ON POISONED WOUNDS,

*delivered at the Philadelphia Medical Institute, by* THOMAS HARRIS, M. D., *Surgeon U. S. N., and to the Pennsylvania Hospital.*

POISONED wounds are among the gravest accidents to which we are liable. Independently of the mechanical injury inflicted by the wound, there is superadded a noxious principle, the effects of which, on the whole system, occasion alarming symptoms, and, not unfrequently, death. These symptoms are more or less violent, according to the character of the deleterious agent, by which they are induced. These agents are derived from the vegetable, mineral, and animal kingdoms.

The poisoned wounds, which chiefly engage our attention, in this country, are those arising from the venom of serpents, and rabid animals. The surgeon is also sometimes called to relieve patients, from the effects of the stings of various insects. Wounds, from the common bee, humble bee, wasp, &c., are sometimes productive of severe inflammation. Death has not unfrequently been the consequence of attacks of these animals. They have a subtle poison, contained in a receptacle, situated within the abdomen, among the air vessels, which is furnished with a proper apparatus, for injecting the wound made by their sting. In some of these insects, this liquor is highly acrimonious, and quickly excites violent inflammation. I believe, in all instances, where death results from the stings of bees or wasps, that it arises from active inflammation, either in or about the larynx, so as to interrupt respiration. I once witnessed the death of a fine horse, from this cause, which had been carelessly hitched in the vicinity of a hive of bees. Upon examining the horse, after death, I found that the bees had ascended his nostrils, as far as the posterior fauces, inflicting upon him stings during their whole course. The consequent inflammation and tumefaction of the tonsils and pharynx, closed the mouth of the larynx, and thus produced suffocation.

The common mosquito, some varieties of fly, spiders, and the scorpion, often inflict severe and dangerous wounds. It is only particular constitutions that suffer severely from the fly or mosquito. Spiders have sometimes caused dangerous wounds, though I believe that most of the species are harmless.

The scorpion is a venomous insect, though I believe its sting does not often prove fatal. In Africa and Persia, where these animals are found of a large size, their wounds sometimes prove fatal, but in the West Indies their sting does not give rise to symptoms more alarming than that from the common honey-bee. The sting of the species found in this country will give rise to slight pain and tumefaction, but is, in other respects, harmless.

Fatal consequences often arise from the bites of serpents. Of the American serpents, two species only are acknowledged to be venomous—the rattle-snake and copper-head. There are several varieties of each of these species. These reptiles are furnished with long and curved teeth, or poisonous fangs. These curved or hooked teeth are pierced by a canal, which terminates at some distance from the point, or the convex part, in a small groove, which extends to the apex of the tooth. The venomous liquor is contained in a sac, at the root of the fang, and under the muscle which depresses the lower jaw. When this muscle acts with violence, as when the animal prepares to bite, it compresses the poisonous reservoir, and throws its contents into the teeth.

The deleterious operation of this poison will materially depend upon its quantity, and upon the season of the year in which the wound is inflicted. The rattle-snake is more lively, and its venom more active during very warm weather, than at any other period. The effects, produced by the poison, vary, according to the parts wounded, the depth to which the fang penetrates, and the quantity and strength of the venom in the reservoir. In some instances, death follows in a few minutes after the injury, and in others not until many days.

The effects are first local and then general. At the moment the bite is inflicted, acute pain is commonly felt, which, in some instances, extends to a considerable distance from the point of injury. The wounded part soon becomes red and tumefied, and not unfrequently these symptoms extend throughout the injured limb, and even to the whole body. At times, blisters are raised about the wound, and a sanious fluid slowly distils from it. Shortly afterwards, the pain greatly diminishes, the limb exhibits an edematous and doughy softness, becomes cold, and the skin is covered with livid and gangrenous spots.

The patient now becomes anxious, respiration is difficult, with great weakness and copious cold sweats; the pulse is depressed, small, and irregular; the countenance ghastly, with muttering delirium.

Vomiting sometimes is an attendant symptom, with copious bilious discharges per anum, cold sweats, and universal yellowness, with severe pains about the umbilicus.

When the effects of the poison are sudden, it is probable that the fang has penetrated a vein, by which the poison was carried directly to the heart. When no serious symptoms have followed the bite of the rattle-snake, it is probable that the teeth entered obliquely and did not entirely penetrate the true skin, or that the poisonous reservoirs were empty, or that the virus itself had become changed, from some cause, in its properties. From these considerations, we may explain why certain articles of the Materia Medica have obtained reputation as antidotes. Nature is, of herself, sometimes enabled to accomplish a cure, or the virus injected has been insufficient in quantity and quality, to produce death.

The copper-head, of this country, is nearly as poisonous as the rattle-snake. The European viper is much dreaded, but according to Fontana, it is a less poisonous reptile, than the snakes of this country, to which I have alluded. The *cobra-de-capello* of India and Africa, is also extremely venomous. A history of their domestication by the natives, as detailed by Bruce, is exceedingly curious.

Wounds from rabid animals are generally alarming, though they do not always produce hydrophobia. It is somewhat remarkable, that such wounds heal as readily, as those inflicted by healthy animals.

A number of persons may be bitten by the rabid animal within a short time of each other, and the wounds may appear equally extensive, and nothing done for their removal, and yet hydrophobia may occur in some cases, and not in others. This difference of effect may arise from some of them being bit through the clothes, by which the venom was wiped off the teeth; from a smaller quantity of the poison being injected into the wound, or from other causes which are still inexplicable. It is a fact, with which we are all familiar, that the system is, at times, in such a state, as to resist all infections or poisons. We see it as regards small-pox, measles, hooping-cough, and, indeed, every other form of contagion or poison, with which the human frame is afflicted. The constitutional disease will sometimes appear, in five or six days after the local injury, and sometimes as many months will elapse before any disturbance of the system becomes apparent. The common period of its appearance, however, is from the thirtieth to the fortieth day. Generally, the wound inflames and becomes painful, before the appearance of the constitutional symptoms; but, sometimes, the disease takes place without any alteration in the site of the wound. When the constitutional disease begins to manifest itself before the wound heals, a remarkable change, in the appearance of the sore, generally takes place. Instead of continuing to granulate and discharge good pus, it inflames, assumes a sloughing appearance, and the discharge becomes ichorous.

Hydrophobia is always preceded by great depression of spirits, a general restlessness, and unhappy state of mind. There is a gloom spread over the countenance, and an inclination to avoid society. It would appear that these primary symptoms do not arise from the alarm excited by the bite of the animal thought to be mad, but are inseparably connected with the disease, as they are also observed to occur in children who have been bitten, and who are incapable of appreciating their danger. To these symptoms succeed a loss of appetite, nausea, a quickness of pulse, a starting in sleep, great thirst, with an inability to swallow liquids. This inability not only gradually increases, but every attempt is attended with inexpressible horror, and universal agitation. There is a perpetual ejection of viscid phlegm from the mouth; the eye now becomes prominent and wild, the countenance anxious, the respiration difficult. At a later period the pulse becomes remarkably quick, with an expression of alarm or apprehension, wild startings, and, sometimes, convulsions, when liquids are presented, or the surface of the body touched with a current of cold air. The operations of the mind are at last so deranged, that the patient becomes completely maniacal. His wild expressions of alarm, and his apparent efforts to escape from the danger by which he imagines himself surrounded, added to his other symptoms, at length produce so much exhaustion, that he sinks, about the fourth or sixth day, under his accumulated sufferings.

There are dangerous injuries, to which *anatomists* are particularly liable, which may, with great propriety, be classed with the poisoned wounds.

Persons, engaged in the dissection or examination of putrid or ulcerated bodies, in macerating or making preparations, have, occasionally, suffered from wounds of the scalpel, or needle, or from spicula of bone.

These wounds give rise to violent inflammation, extending up the arm as high as the axilla, or neck, rendering the whole limb exceedingly painful and much swelled, and, finally, producing extensive abscesses, gangrene, and death. Examples of this kind are by no means uncommon. The celebrated anatomists, Fyfe, Chambon, and Corvisart, all, nearly lost their lives by accidents of this character. Professor Le Clerc, Allan Burns, Dr. Gordon, professor of physiology at Edinburgh, fell victims to wounds from dissection. Some years ago, I attended Dr. P——, of the navy, who wounded himself very slightly by the needle with which he was sewing up a body which we had examined. The patient died from the irritation of an extensive abscess, connected with exostosis. The next day after the occurrence he began to feel pain in the part wounded, which increased in violence, accompanied with tumefaction of the arm and shoulder, fever, delirium, a dark tongue, and tenderness over the epigastric region. With the scalpel, I opened the finger to the bone, and applied a blister, which extended from the hand to the shoulder, and extracted about six ounces of blood, by means of cups, from over the region of the stomach. By such means relief was afforded, and the patient's life was preserved. He was, however, about five or six weeks before he entirely recovered.

The medium through which these various poisons act on the general system, is a point which is still unsettled by pathologists. While some suppose this medium to be the nerves, others the lymphatics, and others, again, the veins; the system may be, and no doubt is, often affected, at times, through each of these several systems. In wounds inflicted in dissecting rooms, and in those arising from certain species of serpents, and from rabid animals, there is sufficient length of time from the infliction of the injury, to the appearance of constitutional symptoms, to allow the poison to enter the system by absorption.

In other cases, however, when death follows the injury in the course of a few minutes, it is more rational to conclude, that the system becomes affected through the agency of the nerves. The bite of the cobra-de-capello produces death in five or six minutes. Life has been destroyed by the external use of prussic acid in a still shorter period. The poison may be conducted, it is true, with great rapidity to the heart by the veins.

The experiments of Magendie satisfactorily establish, what indeed was believed by Celsus and Galen, that *the veins* possess an absorbing power. These experiments have been confirmed by Dr. Barry, an English army surgeon, when resident at Paris.

The object, which this ingenious experimentalist had in view, was to illustrate the "influence exercised by atmospheric pressure, upon the progression of the blood in the veins, upon that function called absorption, and upon the prevention and cure of the symptoms caused by the bites of rabid or venomous animals."

Passing over his speculations on the moving power of the venous blood, I shall briefly notice some of his interesting facts, in relation to poisoned wounds. Presuming that the alarming symptoms, to which these wounds give rise, are occasioned by the absorption of poison into the general circulation, he came to the conclusion that these effects could be prevented by arresting its absorption. This, he thought, might be achieved, provided the "points of contact of the absorbing surfaces, and the substance to be absorbed, were placed under the influence of a vacuum." The removal of the atmospheric pressure was accomplished by means of cupping glasses.

The first knowledge of absorption may be traced in the history of poisoned wounds. The period when the baneful art of inflicting these wounds originated, is lost in the obscurity of fable. The arrows of Hercules, the sufferings and death of Chiron, Nessus, and other fabled personages, afford evidence that a knowledge of these destructive agents, was almost coeval with the history of man. The manner in which poison became mixed with the fluids of the body, was first explained by Celsus. He perceived that the veins were the channels through which the poison was carried into the general system. Hence, if the poisoned wound occurred in one of the extremities, he recommended the application of a ligature above the point of injury.

Hippocrates recommends the application of cups to poisoned wounds, without, however, detailing his own experience of their utility. In the Iliad,

Machaon is made to suck the wound of Menelaus, which is the earliest record we have of a vacuum being applied to a poisoned wound.

When the blood-vessels were pointed out as the channels for transmitting the poison to the system, a ligature above the wound naturally suggested itself, and as cups, or the cucurbitulæ prevented the contents of the veins flowing towards the heart, their utility appeared obvious. Celsus, accordingly, considered that cupping glasses should be almost exclusively relied upon, both as a preventive and remedial agent in cases of poisoned wounds.

He directs that a ligature should first be tied above the wound, then cupping glasses should be applied as the most effectual means of extracting the poison. Direct suction by the mouth was considered, next to cupping, the *best* preventive.

Strato, Pliny, and Plutarch speak of the *Psylli*, the *Marsi*, and the Ophigenes, a class of persons, who were supposed to possess a hereditary power of curing the bites of numerous serpents. They exercise their skill, in wounds of this character, by sucking them. Their great success, in such cases, procured them a reputation, so deservedly high, that they became indispensable appendages to the Roman armies. A corps of these wound-suckers was attached to the army of Cato during his campaigns in Africa. It is stated by Suetonius, that Augustus ordered the Psylli and Marsi to suck the wounds of Cleopatra, with the hope of restoring her to life, after she expired from the bite of an asp.

It is stated on the authority of Camden and Fuller in their history of the Holy Wars, that Queen Eleanor saved the life of King Edward I. of England, by sucking the poison from a wound, inflicted by a Mahometan assassin.

After the discovery of the *lymphatic* system, the lymphatics were long considered as the sole agents of absorption, which was carried on by a vital action, and the treatment of poisoned wounds underwent a sudden revolution. The cupping glasses were laid aside as too mechanical. The lymphatics had taken up the poison by an inherent vital principle. Stimulants must, therefore, be given, to modify their action and to induce the exhalents to throw off the morbid matter. Irritants must be applied to the wound on the principle "ubi stimulus ibi affluxus." The discharge was to be kept up by every possible means, whilst the vitality of the absorbents was to be destroyed by caustics.

Thus did theory, or, what is more commonly dignified by the name of principle, triumph over facts, or over a well established course of practice. It remained for Barry to conduct us back to the point from which we had been enticed by theorists, and to establish, by well contrived and judicious experiments, the utility of a practice, not less distinguished for the antiquity of its origin than for its triumphant efficacy.

To establish the value of creating a vacuum over the poisoned surface, Dr. Barry procured different kinds of poisons, of known activity, such as prussic acid, strychnine, upas, cicuta, arsenic, &c. &c. With these deleterious agents he experimented on rabbits and dogs, having always two animals placed under corresponding circumstances, as regards depth of wound, point of insertion, and quantity of substance employed, *except* that the cupping glass was applied to the one, while the other was consigned to his fate. The neglected animal always died sooner or later, while the one to which the vacuum was applied, never showed the slightest symptoms of poisoning, although the destructive agent remained in contact with the wounded surface during the space of an hour, and even so long as five hours in succession.

These experiments were performed in presence of several distinguished members of the French Institute. The report to that body confirming the truth of Dr. Barry's views was drawn up by the late professor Laennec.

Further, to test the efficacy of the cupping glass, our experimentalist had several dogs and rabbits bitten by vipers. To the bites of some, he applied the cupping glasses, whilst to the others nothing. Although the animals abandoned did not ultimately perish, the results obtained by the comparison were precisely analogous, as far as regards symptoms, to those noticed in his former experiments.

"Animals bitten by one, two, and sometimes by three vipers, on which the cupping glasses had been applied for half an hour, experienced no symptoms of constitutional poisoning, whilst those that were entirely neglected, were invariably attacked with convulsions, stupor, and the dogs by vomiting. Pigeons invariably died from one bite of the viper, the fatal symptoms generally commencing before the end of the fifth minute; but, when the cupping glass was applied immediately after the bite, they showed no signs of having absorbed the poison, while the glass remained on." Even in cases where the poisoned animal had been left to its fate until convulsions or tetanic symptoms had supervened, the application of the cups suspended entirely all the alarming symptoms.

The experiments of Dr. Barry were repeated some years ago, by Dr. Pennock, of this city, and with similar results. These experiments were conducted with great skill and judgment.

During his experimental inquiry, he discovered that pressure, or the application of a weight equal to fifteen pounds over the wounded surface, proved quite as effectual in preventing, as well as in suspending the symptoms of poisoning, as the establishment of a vacuum. The poison employed in this experiment was *strychnine*, the fatal strength of which had been tested in former experiments. It must, therefore, be obvious, that there could be no mistake in Dr. Pennock's observations on this point.

After the removal of the weight, as well as of the cups, the poison has sometimes been observed to excite constitutional symptoms. In some instances such effects have been induced several hours after the cups or weights had been employed. In a practical point of view this fact is of great importance. It teaches us not to rely exclusively in alarming cases on the cupping glass. Though this preventive agent may prove abundantly efficacious, under ordinary circumstances, yet in cases of bites from rabid animals, or in poisons of slow operation, other means should be used, besides those which I have already detailed. These means are such as have been found efficacious in the hands of surgeons in all ages.

The first thing, then, to be done in treating the recent bite of a rabid animal, is to apply a powerful cupping glass over the wound. This measure supersedes, at once, the use of the ligature, ablution, excision, &c., during the period of its application, and for a certain time after its removal. After the cupping glass has been applied for an hour, *at least*, the whole of the parts wounded or abraded by the bite, should be freely dissected out. The application of the cupping glass will extract a portion of the poison, and concentrate to a particular point the remainder of it, so that it can be, generally, removed by excision. Afterwards the cups should be reapplied, in order to remove any of the poison which had been forced into a vessel beyond the reach of the knife.

I believe, with Dr. Barry, that the "notion of hydrophobic poison being absorbed in the usual manner, and that it lurks in the system several weeks before it produces its peculiar effects, is contrary to all analogy." He believes that, in cases of poisoning from the bites of rabid animals, as in all others, the "commencement of the symptoms, is synchronous with the consummation of the absorption, and that their repetition is dependent upon its renewal."

As an evidence that the poison remains inactive and stationary, inflammatory symptoms occur in the injured part a few days previously to the explosion of the constitutional symptoms. These circumstances are replete with practical instruction. They teach us that so soon as the cicatrix grows tender or sore, or when there is sufficient evidence that the animal, which inflicted the wound, was rabid, we should immediately apply the cupping glass, and proceed exactly as in the case of a recent bite. As we have evidence that the cupping glasses removed tetanic symptoms, we should not be deterred from using them even after hydrophobia has commenced.

In cases where the cupping instruments cannot be immediately commanded, suction, by means of the mouth, may be advantageously substituted. When the mouth is free from wounds or ulcers, there is little risk in the experiment. Dr. Benjamin Smith Barton swallowed the poison of the rattlesnake, without suffering any other inconvenience than a slight irritation of the fauces.

Excision alone can be useful only when it reaches beyond the poison. It is stated by Fontana, that the particles of poison which may have been forced further than the boundary of the excised wound, will be sent to the heart with greater rapidity after the operation than it otherwise would have been, owing to the wider mouths of the vessels being now fully exposed to receive the atmosphere within their cavities.

Hence, the necessity of first applying the cups by which a retrograde movement of the fluids is effected and also of their reapplication after the injured part is excised, in order to remove any remaining portion of the poison which may have penetrated beyond the reach of the knife.

If the part bleed freely after these operations, the hæmorrhage may be arrested by the actual cautery. Besides burning the small vessels, their mouths are sealed and rendered incapable of further absorption. I have no doubt that this course of treatment will prove efficacious in every species of poisoned wound, if practised, at a period sufficiently early; no matter whether the wound arise from the sting of insects, an injury in the dissecting room, or from the bite of a serpent or rabid animal.

There are other remedies, which have acquired reputation in the management of poisoned wounds, but it is very difficult to ascertain the degree of reliance, which ought to be placed on them. There are many wounds of this character, which would terminate, favourably, if left to the operations of nature, and there are others, in which no alarming symptoms supervene. When, then, these agents are employed, either as preventives or curatives, it is impossible to say whether the cases would not have done equally well had they not been used. These are points which can only be settled by a series of experiments. Whoever shall take upon himself this trouble will at once subserve the cause of science and of humanity.

These remedies consist of the internal and external use of ammonia; of olive oil; hieraceum venosum, a species of the rattlesnake root; alisma plantago, or water plantain, and many others of scarcely less notoriety.

The records of medicine furnish the strongest testimony in favor of each, but for the reasons which I have already stated, they cannot, and ought not to command our confidence until their utility is established by judicious experiments. This is, more particularly, our duty, since we have a remedy, the efficacy of which is established beyond the reach of doubt.

